# The Effect of Soil Type and Moisture on the Development of Forensically Important *Megaselia scalaris* and *Dohrniphora cornuta* (Diptera: Phoridae)

**DOI:** 10.3390/insects15090666

**Published:** 2024-09-01

**Authors:** Wei Han, Dianxing Feng, Yanan Tang

**Affiliations:** College of Life Science and Engineering, Shenyang University, Shenyang 110044, China; hw06122024@163.com (W.H.); tyn0986@163.com (Y.T.)

**Keywords:** phorid fly, soil moisture, soil texture, post-mortem interval, developmental time

## Abstract

**Simple Summary:**

The study examined the effects of three soil types and six moisture contents on the development of two representative insects found on buried corpses, *Megaselia scalaris* (Loew, 1866) and *Dohrniphora cornuta* (Bigot, 1857) (Diptera: Phoridae). Soil type, soil moisture, and their interactions had significant effects not only on the larval and pupal survival of the two species but also on their development time and larval body length, two common indicators used to infer the time of death. Soil moisture had the greatest effect on the development of both species. Moisture contents of 20% and 40% were more suitable for the survival of two phorid flies in three soils. In addition, it was found that larvae can survive on extremely dry soils (0% moisture), although their development time is longer than in other moisture contents. When using developmental data of phorid flies to infer the post-burial interval (PBI) of buried corpses in forensic investigations, it is crucial to consider the effects of soil type and moisture content to avoid inaccurate estimations.

**Abstract:**

Necrophagous phorid flies are common insects found on buried corpses, and their developmental data play a crucial role in estimating the post-burial interval (PBI). This study aimed to investigate the effects of soil type and moisture content on some life cycle parameters of two forensically important insects, *Megaselia scalaris* (Loew, 1866) and *Dohrniphora cornuta* (Bigot, 1857) (Diptera: Phoridae). Larval and pupal survival, development time, and larval body length of *M. scalaris* and *D. cornuta* were observed in three different soil types (loamy sand, sandy loam A, and sandy loam B) with six moisture contents (0%, 20%, 40%, 60%, 80%, and 100%). The results indicated that soil types, soil moisture, and their interaction significantly influenced the growth and development of both species, with moisture being the most influential factor. In each soil, 20% and 40% moisture contents were more suitable for their growth and development. Both the development time and maximum larval body length were significantly different among soil types and moisture contents. The larval period of both species lasted the longest in all soils with 0% moisture content. Additionally, a regression analysis of the relationship between larval body length and development time was performed at different moisture contents in three soils. This study expanded our knowledge of the factors that influence the development of necrophagous insects and provided some reference data for applications of *M. scalaris* and *D. cornuta* in PBI estimation.

## 1. Introduction

Necrophagous flies can reach a corpse within minutes after death and deposit eggs or larvae. The first instar larvae that hatch from the eggs feed on the corpse and continue to grow. Therefore, the post-mortem interval (PMI) of the corpse can be determined indirectly from the developmental data of the flies [1,2].

Since insects are poikilothermic, temperature has a great influence on their growth and development. Developmental data have been established for many insect species of importance in forensic entomology [3,4,5,6,7,8,9,10,11,12,13]. However, previous studies have shown that the soil moisture content and soil type play a significant role in the development of larvae and pupae of some insects that have soil-pupation behaviors [14,15,16,17,18,19,20,21,22,23,24,25,26]. For example, Alyokhin et al. [14] found that *Bactrocera dorsalis* (Hendel, 1912) preferred to pupate in soils with higher moisture and larger particles. Hou et al. [15] reported that extremely wet conditions negatively affected the pupal survival and emergence of *B. dorsalis*. Chen and Shelton [17] showed that both soil moisture and pupation depth influenced the emergence of *Contarinia nasturtii* (Kieffer, 1888), but the influence of soil type was not significant. Amaral et al. [22] found that soil class was the most important variable for pupal development in *Bactrocera carambolae* (Drew and Hancock, 1994). Moisture content had no significant effect on pupation depth or pupal emergence. Kökdener and Şahin Yurtgan [24] showed that soil type and moisture content had a significant effect on the development time and larval and pupal survival of *Lucilia sericata* (Meigen, 1826). Pan et al. [25] reported that soil type and moisture content significantly affected the emergence of *Frankliniella intonsa* (Trybom, 1895), but had no significant effect on the development time. The emergence rate of *F. intonsa* initially increased and then decreased with increasing soil moisture content in all tested soils.

Compared to middle–large-sized necrophagous flies such as blow fly (Diptera: Calliphoridae) and flesh fly (Diptera: Sarcophagidae), the phorid fly (Diptera: Phoridae) is tiny and can easily enter the room through cracks in doors and windows [27,28] or pass through soil gaps to reach the carcass [29,30,31,32], becoming a representative insect in indoor or buried carcasses. When phorid flies penetrate the soil to colonize the buried carcasses, they can complete their entire life cycle in the soil and even reproduce for several generations [33]. Since they are poikilotherms, there is no doubt that soil temperature has an impact on their development, and whether other soil environmental factors such as soil moisture and soil type also have an impact on their development is unknown.

Thus, *Megaselia scalaris* (Loew, 1866) and *Dohrniphora cornuta* (Bigot, 1857), two forensically important phorid flies that were recovered from buried human corpses [34,35,36,37], were used in this study. The survival and development duration of the larvae and pupae, as well as the body length of the larvae of the two species, were measured at six moisture levels (0%, 20%, 40%, 60%, 80%, and 100%) in three soils (loamy sand, sandy loam A, and sandy loam B) to evaluate the effect of soil type and moisture on their growth and development.

## 2. Materials and Methods

### 2.1. Insects

*Megaselia scalaris* and *D. cornuta* were obtained from the laboratory colony of Liaoning Key Laboratory of Urban Integrated Pest Management and Ecological Security, Shenyang University, China. The two laboratory colonies established from adult specimens, baited using pork in Shenyang City, Liaoning Province, China, were reared with lean pork for over 10 generations at 21–24 °C, 75% relative humidity, and a 12 L/12 D photoperiod.

### 2.2. Soils

Loamy sand, sandy loam A, and sandy loam B were used in this study. Loamy sand and sandy loam A were collected from Shenyang City, Liaoning Province, and sandy loam B from Shijiazhuang City, Hebei Province. The characteristics of each soil were examined by the Key Laboratory of Eco-restoration of the Regional Contaminated Environment of the Ministry of Education (College of Environment, Shenyang University) (Table 1). Each soil sample was sieved to remove plant debris and stones and then completely dried in an oven at 80 °C for 5–6 h. The soil moisture content was calculated according to the formula provided by Chen and Shelton [17] as below:

Soil moisture (%) = [weight of distilled water added/(weight of saturated soil − weight of dry soil)] × 100%

The soil was divided into 6 different moisture contents: 0%, 20%, 40%, 60%, 80%, and 100%, and 0% moisture content was completely dry soil.

### 2.3. Observation on the Development of Necrophagous Phorid Flies

Five to ten pairs of adult flies of both species were maintained in a 1000 mL narrow-necked bottle (Sichuan Shubo Co., Ltd., Chongzhou, China) sealed with an industrial filter cloth (Suzhou Tebang Environmental Protection Technology Co., Ltd., Suzhou, China) and fed with fresh lean pork in an artificial climate incubator (Ningbo Laifu Technology Co., Ltd., Ningbo, China) at 27 °C, 75% relative humidity, and a 12 L/12 D photoperiod. The eggs were removed from the bottles and placed in a petri dish with a little distilled water at the bottom. The petri dish was sealed with parafilm and then transferred to an artificial climate incubator set at 27 °C, 75% relative humidity, and a photoperiod of 12:12 (L/D) h, and the eggs were regularly examined for hatching. After egg hatching, the first instar larvae were carefully brushed out and placed on fresh lean pork. Collections continued for 30 min. Therefore, time zero was egg hatching + 30 min max. The prepupae were picked from the bottles after post-feeding larval pupariation. Collections continued for 1 h. Time zero was therefore prepupa formation + 1 h max. The first instar larvae, along with lean pork or prepupae were then transferred to clear plastic bowls (diameter of upper side: 11.8 cm, diameter of bottom side: 6.8 cm, height: 6.5 cm) containing different soils with different moisture contents and were buried in the soils. A plastic lid was placed on each plastic bowl to prevent the evaporation of soil water. The bowls were placed in an artificial climate incubator at a temperature of 27 °C, relative humidity of 75%, and a photoperiod of L/D = 12 h/12 h. The development of the larvae and pupae was observed regularly. There were 10 replicates for each test. The larval feeding period was recorded from the egg hatch to the first larva leaving the pork tissue. The larval period was recorded from the egg hatch to the first larva pupation. The pupal period was recorded from prepupa to the first adult emergence. Pupation rate and emergence rate were calculated by the following formula:

Pupation rate (%) = (Number that pupated normally/Total number of tested larvae) × 100%

Emergence rate (%) = (Number of adults emerged/Total number of tested pupae) × 100%

### 2.4. Measurement of Larval Body Length

After the eggs hatched, ten larvae were randomly sampled every 6 h until the first larva started pupariation. The sampled larvae were killed with hot water and stored in a 75% alcohol solution. The larvae were placed under an Olympus BX41 stereomicroscope. Pictures were taken using an Olympus DP-71 digital camera and DP Controller 3.1 software. The body length of the larvae in each picture was measured using the measurement tool in Image-ProPlus 6.0 software.

### 2.5. Statistical Analysis

The data analysis was performed using SPSS 27.0 and Graphpad Prism 9.5 software. The effects of the soil moisture and soil type were analyzed using a two-way analysis of variance (ANOVA) test. The normality of the distribution and homogeneity of variance were tested using the Shapiro–Wilk normality test and Levene’s test, respectively. The larval and pupal development time and larval body length between different moisture contents in each soil and between different soils at each moisture content were compared using a one-way ANOVA, followed by Tukey’s Honestly Significant Difference test. In all tests, significance levels were determined at α = 0.05. The relationships between larval body length and development time after hatching were determined by regression analyses.

## 3. Results

### 3.1. Effects of Soil Type and Moisture on the Survival of Larvae and Pupae

The pupation rate and emergence rate of *M. scalaris* and *D. cornuta* in three soils with different moisture contents are shown in Table 2 and Table 3. In the three soils, the larvae of both species were able to develop and pupate at 0–60% moisture. Their pupae were able to emerge normally at moisture levels between 20% and 60%. At 0% moisture, the pupae shrank and died. In addition, both species could also pupate and emerge in loamy sand with a moisture content of 80% (Table 2 and Table 3).

Both species had the lowest pupation rate at 0% moisture content in the three soils: for *M. scalaris*, it was 51.34 ± 0.08% in loamy sand, 43.67 ± 0.07% in sandy loam A, and 53.00 ± 0.03% in sandy loam B; for *D. cornuta*, it was 57.00 ± 0.05% in loamy sand, 24.34 ± 0.04% in sandy loam A, and 41.67 ± 0.07% in sandy loam B. The highest pupation rate and emergence rate of the two species were observed at 20–40% moisture content in each soil. The lowest emergence rates of both species were in sandy loam A with 60% moisture content. The lowest emergence rates were 46.50 ± 0.06% for *M. scalaris* and 36.00 ± 0.06% for *D. cornuta*.

Soil type, soil moisture, and soil type × soil moisture had significant effects on the survival of larvae and pupae of the two species (Table 4 and Table 5). By analyzing the contribution rate of each factor, it was found that soil moisture had the greatest influence on the pupation and emergence of the two species.

### 3.2. Effects of Soil Type and Moisture on the Development Duration of Larvae and Pupae

**Figure 1:**
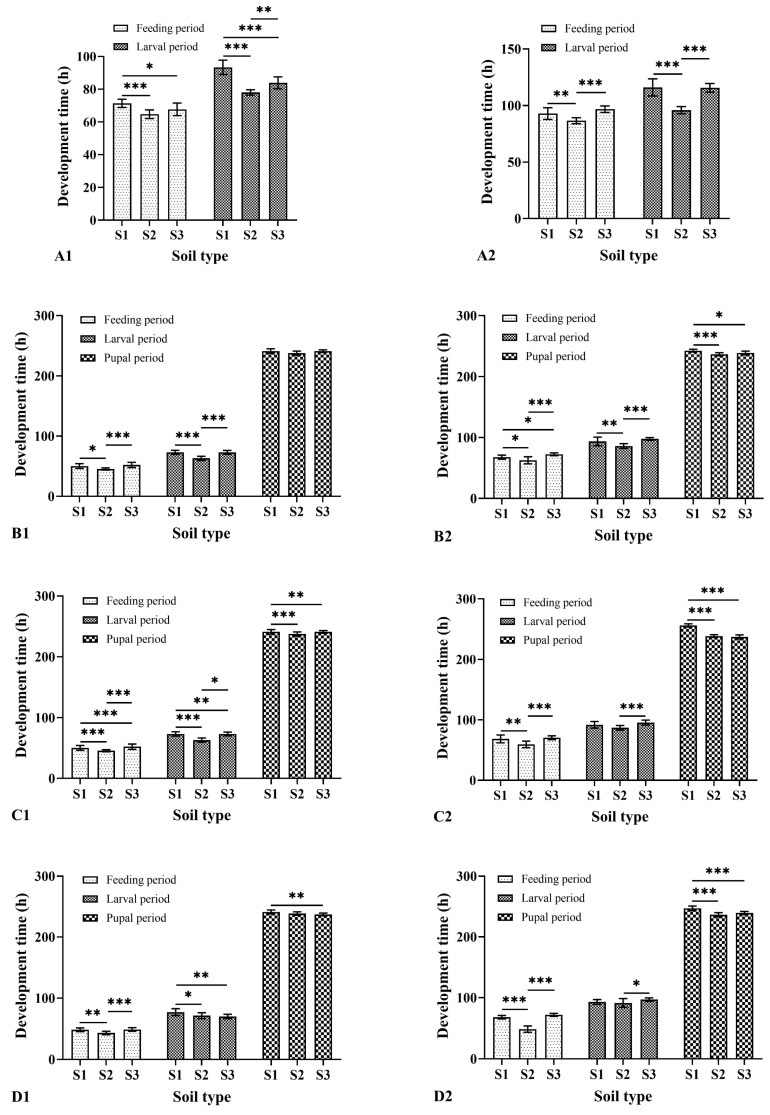
Development time of *Megaselia scalaris* and *Dohrniphora cornuta* in three soils with different moisture contents. 1: *Megaselia scalaris*; 2: *Dohrniphora cornuta*. (**A**): 0% moisture; (**B**): 20% moisture; (**C**): 40% moisture; (**D**): 60% moisture. S1: loamy sand; S2: sandy loam A; S3: sandy loam B. * represents *p* < 0.05, ** represents *p* < 0.01, *** represents *p* < 0.001.

There were significant differences in the duration of the larval feeding period, larval period, and pupal period of the two species among soil type and soil moisture (Table 2 and Table 3, Figure 1). The larval period of both species lasted the longest in all three soils at 0% moisture content. *M. scalaris* was 93.33 ± 4.37 h in loamy sand, 77.98 ± 1.69 h in sandy loam A, and 83.90 ± 3.68 h in sandy loam B, whereas *D. cornuta* was 115.98 ± 7.61 h in loamy sand, 95.85 ± 3.18 h in sandy loam A, and 115.65 ± 3.81 h in sandy loam B. In both species, the longest duration of the pupal period occurred in loamy sand. The longest pupal development duration of *M. scalaris* was 248.73 ± 3.10 h at 80% moisture, while *D. cornuta* was 256.23 ± 2.67 h at 40% moisture.

By analyzing the contribution rate of soil type, moisture, and their interaction, it was found that soil moisture had the greatest influence on the duration of the larval feeding period, larval period, and pupal period of the two species (Table 4 and Table 5).

### 3.3. Effects of Soil Type and Moisture on the Larval Body Length

**Figure 2:**
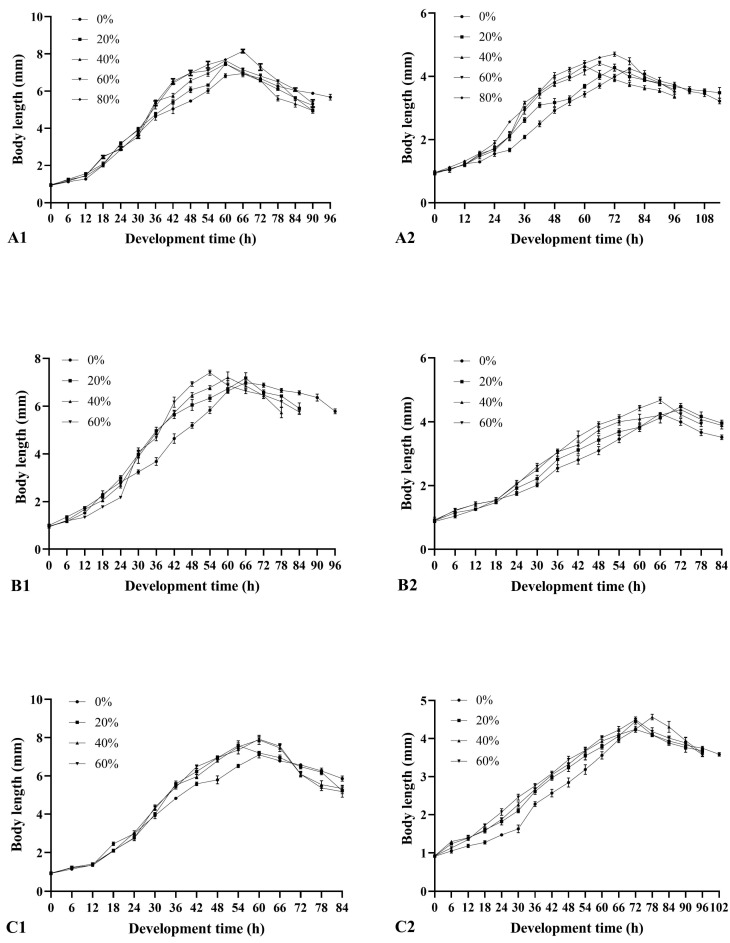
Larval body length changes of *Megaselia scalaris* and *Dohrniphora cornuta* in three soils with different moisture contents. 1: *Megaselia scalaris*; 2: *Dohrniphora cornuta*. (**A**): loamy sand; (**B**): sandy loam A; (**C**): sandy loam B.

**Figure 3:**
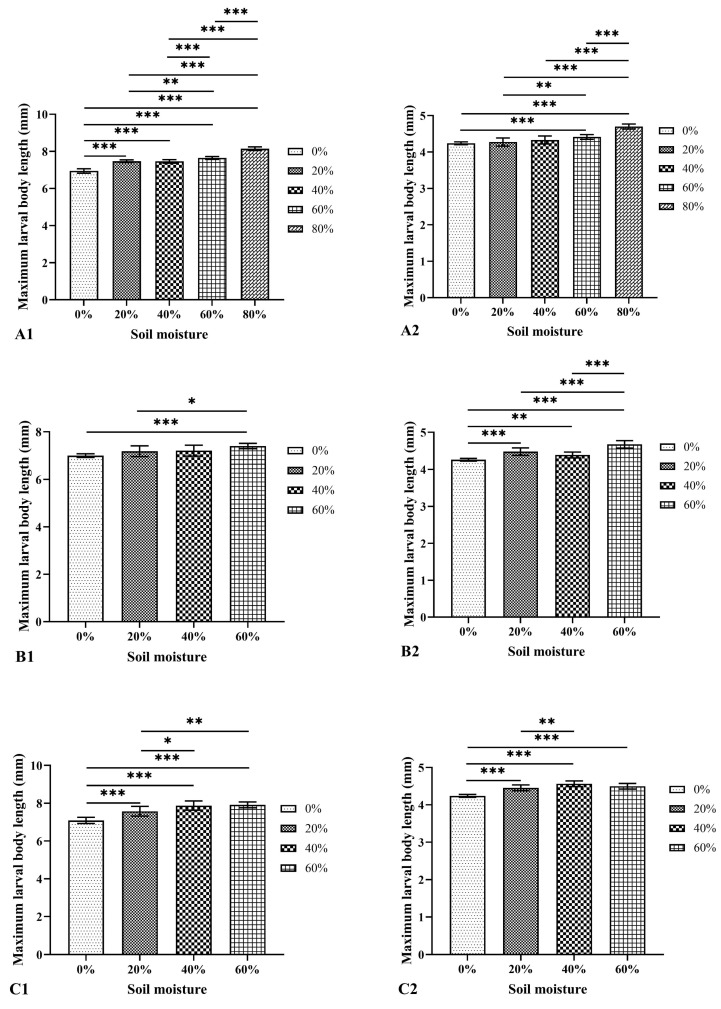
Maximum larval body length of *Megaselia scalaris* and *Dohrniphora cornuta* in each soil with different moisture content. 1: *Megaselia scalaris*; 2: *Dohrniphora cornuta*. (**A**): loamy sand; (**B**): sandy loam A; (**C**): sandy loam B. * represents *p* < 0.05, ** represents *p* < 0.01, *** represents *p* < 0.001.

**Figure 4:**
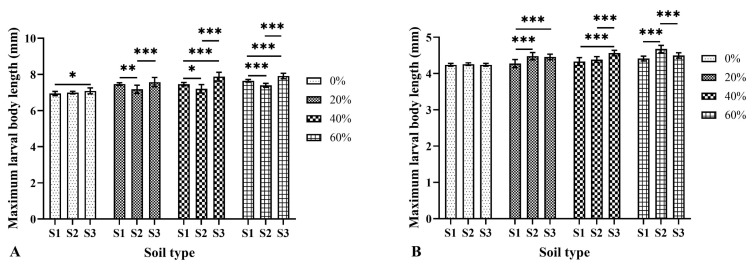
Maximum larval body length of two phorid flies in different soils with the same moisture content. (**A**): *Megaselia scalaris*; (**B**): *Dohrniphora cornuta.* S1: loamy sand; S2: sandy loam A; S3: sandy loam B. * represents *p* < 0.05, ** represents *p* < 0.01, *** represents *p* < 0.001.

In three soils with a moisture content of 0–80%, the larval body length of *M. scalaris* and *D. cornuta* increased continuously over time and then shortened after reaching the maximum (Figure 2). There were significant differences in the maximum average larval body length of both species at different moisture contents in each soil type (Figure 3). The maximum average larval body length of *M. scalaris* (8.15 ± 0.10 mm at 66 h, 80% moisture) and *D. cornuta* (4.70 ± 0.07 mm at 72 h, 80% moisture) was observed in loamy sand. At the same moisture content, the maximum average larval body length of *M. scalaris* varied significantly among different soils, and that of *D. cornuta* also showed significant differences at moisture contents other than 0% (Figure 4). Soil moisture had the greatest effect on the maximum average larval body length of the two species by analyzing the contribution rate of soil type, moisture, and their interaction (Table 4 and Table 5).

Regression equations were determined for the larval body length (mm) and development time after hatching (hours) of both species using larval body length as the independent variable and development time as the dependent variable (Table 6 and Table 7). There was a cubic curve relationship between these two variables in three soils with different moisture contents.

## 4. Discussion

To date, there is no other published research examining the influence of soil type and soil moisture on phorid fly development. Previous studies have shown that soil texture has a significant effect on insects that need to pupate in the soil, such as *Longitarsus bethae* (Savini and Escalona, 2005) [18], *B. carambolae* [22], *L. sericata* [24], *F. intonsa* [25], *Anastrepha ludens* (Loew, 1873), and *Anastrepha obliqua* (Macquart, 1835) (Diptera: Tephritidae) [38]. Eskafi and Fernandez [39] found that soil texture was the primary factor influencing the pupal survival of *Ceratitis capitata* (Wiedemann, 1824), while Bento et al. [40] reported that the moisture in the soil had an impact on the emergence of *C. capitata*. Amaral et al. [22] demonstrated that soil type had a significant impact on *B. carambolae* emergence, but the emergence rate was not significantly correlated with moisture alone. Pan et al. [25] found that the highest emergence rate of *F. intonsa* was higher in sandy soil than in loamy soil and clay soil, confirming that sand content in soils affected the emergence of *F. intonsa*. However, our results showed that soil type, soil moisture, and their interaction had a significant impact on pupation and emergence, with soil moisture having the greatest influence.

For both species, the pupation rate of larvae was the lowest at 0% moisture in the three soils, and their pupae failed to develop into adults at this moisture level. The highest pupation rate and emergence rate of these two phorid flies were recorded at a moisture content of 20–40%. Compared to a 20–40% moisture content, the pupation rate and emergence rate of both species decreased at a 60–80% moisture content, and they could not develop normally at a 100% moisture content. Similar to our results, Li et al. [21] reported that the optimal soil moisture content for pupation of *Mythimna separata* (Walker, 1865) was 20–40%, and 0% and 100% soil moisture were not suitable for pupation. Yan et al. [41] reported that the soil moisture was 40% to 60%, which had a positive effect on the emergence of *Spodoptera frugiperda* (Smith and Abbot, 1797). The moisture content of the soil was too low (<20%) and too high (>80%), which was not conducive to the emergence of *S. frugiperda*. Dry soil causes water loss in insects, resulting in death [20,42], and when the soil is completely saturated with water, it leads to a reduction in oxygen levels in the soil, which inhibits the development of insects [20,23,39]. Eskafi and Fernandez [39] showed that *C. capitata* had higher larval and pupal mortality in 0% moisture and high-density soils. Hulthen and Clarke [16] also reported that 0% soil moisture caused 85% pupal mortality in *Bactrocera tryoni* (Froggatt, 1897). However, Kökdener and Şahin Yurtgan [24] reported that, in contrast to our results, the lowest viability rates of *L. sericata* occurred in soil samples with high moisture contents (100%). In addition, in our study, the pupation rate and emergence rate of *D. cornuta* were much lower than those of *M. scalaris* in sandy loam A with 0% and 60% moisture content, but higher in sandy loam B with 60% moisture content. Genetic differences in Phoridae species may contribute to the different pupation and emergence rates of these two phorid flies.

The development time of immature necrophagous insects is one of the most commonly used indicators of PMI inference in forensic practice [1,2]. In this study, soil type and soil moisture also had a significant effect on the development time of these two phorid flies. There were significant differences in the larval development time of the two species at different moisture contents in three soils. This was also the case for another important forensic insect, *L. sericata*, but the larval development time of *L. sericata* increased with increasing soil moisture content. The larvae were able to survive in soil with high moisture (75–100%), but the development time was longer than that at other moisture levels [24]. However, in our study, the larval development time of *M. scalaris* and *D. cornuta* was the longest in all three soils with 0% moisture content. At 100% moisture, both species could not survive in the three soils.

Zuha and Omar [6] reported that at 27 °C and 50–60% relative humidity, the development time of *M. scalaris* was 76.00 ± 0.00 h for larvae and 180.00 ± 6.90 h for pupae. However, in our study, the larval and pupal development time of *M. scalaris* was 63.04 ± 3.45 h and 237.57 ± 3.41 h, respectively, at 27 °C and 20% moisture in sandy loam A, where this species had a high survival rate. Wu et al. [43] reported that the larval development time of *D. cornuta* at 27 °C and 75% relative humidity was 145.26 ± 2.31 h. In our study, at 27 °C and 20% moisture in sandy loam A, the development time of *D. cornuta* larvae was 86.04 ± 3.91 h, which was significantly shorter than that reported by Wu et al. [43]. So, when using phorid flies to infer the PBI of buried corpses, their developmental data constructed at different constant temperatures and humidity cannot be easily applied, which may lead to an incorrect PBI estimate.

Usually, temperature can significantly affect the development time of necrophagous flies and the body length of their larvae [44,45]. The results of this study showed that soil type and soil moisture could not only affect the duration of the development period but also larval body length. At the same moisture content, the maximum larval body length of *M. scalaris* varied significantly in different soils. The maximum body length increased with increasing moisture content and ranged from 6.94 ± 0.12 mm (0% moisture, loamy sand) to 8.15 ± 0.10 mm (80% moisture, loamy sand). In contrast, Zuha and Omar [6] reported 4.64 ± 0.77 mm at 27 °C and 50–60% relative humidity in Malaysia. In *D. cornuta*, the larvae took 60–78 h to reach the maximum body length, which ranged from 4.24 ± 0.06 mm (0% moisture, loamy sand) to 4.70 ± 0.07 mm (80% moisture, loamy sand), different from that (4.89 mm) reported by Wu et al. [43] at 27 °C and 75% relative humidity. These differences in the development of both species across studies may be due to differences in population genetics and experiment design.

In forensic entomology, larval body length and development time are the most commonly used indicators for PMI estimation [1,2]. Soil type and moisture had significant effects on both indicators. Therefore, in the future, it is necessary to obtain developmental data on necrophagous phorid flies not only at different temperatures and relative humidities but also at different temperatures and moisture contents in different soils to meet the need for PMI inference of corpses emerging in different environments.

## 5. Conclusions

In this study, the effects of soil type, soil moisture, and their interaction on larval and pupal survival, duration of developmental period, and larval body length of *M. scalaris* and *D. cornuta* were significant, with soil moisture having the greatest effect. Extremely dry (0% moisture) and wet (100% moisture) soils had a negative effect on the development of larvae and pupae of these two phorid flies. Moisture contents of 20% and 40% were more suitable for the growth and development of both species. Both the duration of the development period and the maximum larval body length were significantly different among the different soil types and soil moisture levels. In forensic practice, when using developmental data from Phoridae to infer the PBI of buried bodies, it is very important to consider the effects of soil type and moisture on their development to avoid making inaccurate estimations.

## Figures and Tables

**Table 1:** Characteristics of soil used in this study.

Soil	Site	Particle Size (%)			pH	Organic Matter (%)
		Sand	Silt	Clay		
Loamy sand	123°7′31″ E, 41°38′39″ N	83.03	15.79	1.17	6.50	0.15
Sandy loam A	123°26′49″ E, 41°54′39″ N	49.01	48.24	2.75	6.01	1.77
Sandy loam B	114°13′51″ E, 38°29′8″ N	58.67	39.16	2.17	5.05	1.13

**Table 2:** Development time (h), pupation rate, and emergence rate of *Megaselia scalaris* in three soils with different moisture levels (mean ± SD).

Soil	Moisture (%)	Feeding Period	Larval Period	Pupal Period	Pupation Rate (%)	Emergence Rate (%)
Loamy sand	0	71.37 ± 2.47 c	93.33 ± 4.37 c	-	51.34 ± 0.08 a	-
	20	50.22 ± 4.04 a	73.03 ± 3.52 ab	241.05 ± 3.82 a	91.00 ± 0.08 c	93.50 ± 0.05 c
	40	57.60 ± 2.90 b	77.00 ± 4.66 b	244.57 ± 4.10 a	86.67 ± 0.06 c	80.50 ± 0.07 b
	60	48.27 ± 2.99 a	76.96 ± 5.66 b	241.17 ± 3.30 a	86.34 ± 0.07 c	68.17 ± 0.09 a
	80	48.31 ± 2.05 a	70.53 ± 2.76 a	248.73 ± 3.10 b	75.33 ± 0.08 b	60.00 ± 0.11 a
	100	-	-	-	-	-
Sandy loam A	0	64.70 ± 2.63 b	77.98 ± 1.69 c	-	43.67 ± 0.07 a	-
	20	45.47 ± 1.74 a	63.04 ± 3.45 a	237.57 ± 3.41 ab	92.01 ± 0.04 c	81.50 ± 0.09 b
	40	42.79 ± 2.59 a	66.12 ± 3.83 a	234.78 ± 3.85 a	86.65 ± 0.08 c	85.50 ± 0.08 b
	60	43.11 ± 2.82 a	71.26 ± 4.76 b	238.52 ± 2.73 b	72.33 ± 0.06 b	46.50 ± 0.06 a
	80	-	-	-	-	-
	100	-	-	-	-	-
Sandy loam B	0	67.68 ± 3.84 b	83.90 ± 3.68 b	-	53.00 ± 0.03 a	-
	20	52.19 ± 4.32 a	73.17 ± 3.14 a	241.00 ± 2.15 b	91.33 ± 0.05 c	94.50 ± 0.04 b
	40	50.36 ± 2.59 a	70.93 ± 2.55 a	237.55 ± 4.84 ab	93.32 ± 0.04 c	92.00 ± 0.04 b
	60	48.80 ± 2.96 a	70.02 ± 3.57 a	237.02 ± 2.29 a	76.67 ± 0.05 b	77.50 ± 0.05 a
	80	-	-	-	-	-
	100	-	-	-	-	-

(1) Different letters indicate significant differences (*p* < 0.05); (2) “-” represents no data.

**Table 3:** Development time (h), pupation rate, and emergence rate of *Dohrniphora cornuta* in three soils with different moisture levels (mean ± SD).

Soil	Moisture (%)	Feeding Period	Larval Period	Pupal Period	Pupation Rate (%)	Emergence Rate (%)
Loamy sand	0	92.87 ± 5.15 b	115.98 ± 7.61 b	-	57.00 ± 0.05 a	-
	20	67.76 ± 3.41 a	93.57 ± 7.07 a	242.47 ± 2.29 a	90.65 ± 0.06 c	90.00 ± 0.05 d
	40	68.57 ± 6.42 a	91.86 ± 5.50 a	256.23 ± 2.67 d	89.99 ± 0.08 c	79.00 ± 0.04 c
	60	68.05 ± 2.85 a	93.01 ± 3.89 a	246.97 ± 3.79 b	91.67 ± 0.05 c	71.50 ± 0.03 b
	80	68.79 ± 3.99 a	89.97 ± 7.04 a	250.42 ± 1.66 c	70.01 ± 0.06 b	60.50 ± 0.06 a
	100	-	-	-	-	-
Sandy loam A	0	86.57 ± 2.64 c	95.85 ± 3.18 b	-	24.34 ± 0.04 a	-
	20	62.53 ± 5.80 b	86.04 ± 3.91 a	236.52 ± 2.66 a	90.00 ± 0.05 c	88.00 ± 0.08 b
	40	59.48 ± 5.25 b	86.88 ± 3.88 a	238.41 ± 2.29 a	83.00 ± 0.07 c	83.50 ± 0.08 b
	60	48.58 ± 5.12 a	91.33 ± 7.08 ab	236.28 ± 3.53 a	41.34 ± 0.09 b	36.00 ± 0.06 a
	80	-	-	-	-	-
	100	-	-	-	-	-
Sandy loam B	0	96.74 ± 2.86 b	115.65 ± 3.81 b	-	41.67 ± 0.07 a	-
	20	72.47 ± 2.32 a	97.62 ± 2.19 a	238.73 ± 3.00 a	94.00 ± 0.05 b	88.50 ± 0.06 ab
	40	70.38 ± 3.22 a	95.60 ± 3.96 a	237.08 ± 3.44 a	93.00 ± 0.05 b	92.50 ± 0.08 b
	60	71.87 ± 2.33 a	96.85 ± 2.86 a	239.18 ± 2.61 a	91.00 ± 0.06 b	83.00 ± 0.05 a
	80	-	-	-	-	-
	100	-	-	-	-	-

(1) Different letters indicate significant differences (*p* < 0.05); (2) “-” represents no data.

**Table 4:** A two-way ANOVA of pupation rate, emergence rate, development time, and larval body length of *Megaselia scalaris*.

Analysis Indicator	Factor	SS_Factor_	*df*	Mean Square	*F*	*p*	Contribution Rate (%)
Pupation rate	Soil type	0.068	2	0.034	8.806	<0.001	1.92
	Soil moisture	3.362	4	0.840	217.573	<0.001	95.05
	Soil type × soil moisture	0.115	6	0.019	4.944	<0.001	3.25
	Error	0.452	117	0.004	-	-	-
	Total	3.989	129	-	-	-	-
Emergence rate	Soil type	0.321	2	0.160	40.075	<0.001	1.98
	Soil moisture	15.548	4	3.887	971.335	<0.001	95.83
	Soil type × soil moisture	0.356	6	0.059	14.840	<0.001	2.19
	Error	0.468	117	0.004	-	-	-
	Total	16.693	129	-	-	-	-
Feeding period	Soil type	1319.645	2	659.822	73.041	<0.001	12.60
	Soil moisture	9046.119	4	2261.530	250.346	<0.001	86.40
	Soil type × soil moisture	436.876	6	72.813	8.060	<0.001	4.17
	Error	1056.932	117	9.034	-	-	-
	Total	11,527.281	129	-	-	-	-
Larval period	Soil type	2198.321	2	1099.160	76.198	<0.001	30.05
	Soil moisture	5142.241	4	1285.560	89.121	<0.001	70.28
	Soil type × soil moisture	542.140	6	90.357	6.264	<0.001	7.41
	Error	1687.721	117	14.425	-	-	-
	Total	9004.142	129	-	-	-	-
Pupal period	Soil type	334.408	2	167.204	18.250	<0.001	0.03
	Soil moisture	1,323,844.856	4	330,961.214	36,123.271	<0.001	99.32
	Soil type × soil moisture	342.404	6	57.067	6.229	<0.001	0.03
	Error	1071.953	117	9.162	-	-	-
	Total	1,333,970.485	129	-	-	-	-
Maximum larval body length	Soil type	3.547	2	1.773	67.537	<0.001	21.25
	Soil moisture	11.609	4	2.902	110.532	<0.001	69.55
	Soil type × soil moisture	1.031	6	0.172	6.545	<0.001	6.18
	Error	3.072	117	0.026	-	-	-
	Total	19.764	129	-	-	-	-

Contribution rate (%) = SS_Factor_ × 100%/(SS_Total_ − SS_Error_) [25].

**Table 5:** Two-way ANOVA analysis of pupation rate, emergence rate, development time, and larval body length of *Dohrniphora cornuta*.

Analysis Indicator	Factor	SS_Factor_	*df*	Mean Square	*F*	*p*	Contribution Rate (%)
Pupation rate	Soil type	1.239	2	0.619	165.633	<0.001	17.42
	Soil moisture	4.958	4	1.239	331.497	<0.001	69.70
	Soil type × soil moisture	1.023	6	0.171	45.623	<0.001	14.38
	Error	0.437	117	0.004	-	-	-
	Total	7.550	129	-	-	-	-
Emergence rate	Soil type	0.403	2	0.201	71.567	<0.001	2.44
	Soil moisture	15.201	4	3.800	1350.396	<0.001	92.13
	Soil type × soil moisture	0.894	6	0.149	52.970	<0.001	5.42
	Error	0.329	117	0.003	-	-	-
	Total	16.828	129	-	-	-	-
Feeding period	Soil type	3963.955	2	1981.977	113.408	<0.001	18.76
	Soil moisture	16,446.456	4	4111.614	235.264	<0.001	77.83
	Soil type × soil moisture	860.151	6	143.358	8.203	<0.001	4.07
	Error	2044.759	117	17.477	-	-	-
	Total	23,176.072	129	-	-	-	-
Larval period	Soil type	2821.122	2	1410.561	54.517	<0.001	26.59
	Soil moisture	6899.243	4	1724.811	66.662	<0.001	65.02
	Soil type × soil moisture	1070.054	6	178.342	6.893	<0.001	10.09
	Error	3027.257	117	25.874	-	-	-
	Total	13,637.478	129	-	-	-	-
Pupal period	Soil type	1786.091	2	893.045	141.661	<0.001	0.13
	Soil moisture	1,343,381.128	4	335,845.282	53,274.138	<0.001	98.91
	Soil type × soil moisture	1293.651	6	215.608	34.201	<0.001	0.10
	Error	737.579	117	6.304	-	-	-
	Total	1,358,925.937	129	-	-	-	-
Maximum larval body length	Soil type	0.452	2	0.226	35.786	<0.001	15.29
	Soil moisture	2.411	4	0.603	95.555	<0.001	81.56
	Soil type × soil moisture	0.457	6	0.076	12.080	<0.001	15.46
	Error	0.738	117	0.006	-	-	-
	Total	3.694	129	-	-	-	-

Contribution rate (%) = SS_Factor_ × 100%/(SS_Total_ − SS_Error_) [25].

**Table 6:** Equations of the relationship between the body length of *Megaselia scalaris* larvae and the time at different moisture contents in three soils.

Soil	Moisture	Equation	R^2^	*F*	*p*
Loamy sand	0%	Y= −0.942X^3^ + 11.227X^2^ − 25.707X + 23.119	0.798	218.798	<0.001
	20%	Y= −0.681X^3^ + 7.429X^2^ − 10.562X + 8.489	0.745	151.552	<0.001
	40%	Y= −0.642X^3^ + 6.114X^2^ − 2.661X − 0.016	0.709	126.867	<0.001
	60%	Y= −0.786X^3^ + 8.457X^2^ − 13.258X + 10.808	0.733	142.508	<0.001
	80%	Y= −0.371X^3^ + 3.528X^2^ + 3.365X − 3.770	0.735	143.965	<0.001
Sandy loam A	0%	Y= −0.628X^3^ + 7.315X^2^ − 11.195X + 9.749	0.861	342.223	<0.001
	20%	Y= −0.127X^3^ + 1.772X^2^ + 3.969X − 2.196	0.844	264.044	<0.001
	40%	Y= −0.162X^3^ + 1.527X^2^ + 6.661X − 4.239	0.844	246.156	<0.001
	60%	Y= −0.715X^3^ + 7.826X^2^ − 13.198X + 14.000	0.800	194.816	<0.001
Sandy loam B	0%	Y= −0.396X^3^ + 4.951X^2^ − 6.778X + 7.786	0.842	259.906	<0.001
	20%	Y= −0.467X^3^ + 4.530X^2^ − 0.334X + 0.429	0.757	151.967	<0.001
	40%	Y= −0.341X^3^ + 3.228X^2^ + 3.202X − 2.026	0.732	133.169	<0.001
	60%	Y= −0.194X^3^ + 0.932X^2^ + 13.204X − 11.438	0.715	122.336	<0.001

**Table 7:** Equations of the relationship between the body length of *Dohrniphora cornuta* larvae and the time at different moisture contents in three soils.

Soil	Moisture	Equation	R^2^	*F*	*p*
Loamy sand	0%	Y= −5.818X^3^ + 40.085X^2^ − 52.761X + 25.961	0.799	260.359	<0.001
	20%	Y= −7.410X^3^ + 56.258X^2^ − 100.740X + 59.603	0.765	213.046	<0.001
	40%	Y= −4.718X^3^ + 30.775X^2^ − 34.325X + 13.267	0.744	161.197	<0.001
	60%	Y= −2.499X^3^ + 17.586X^2^ − 14.257X + 5.266	0.780	196.268	<0.001
	80%	Y= −3.026X^3^ + 22.684X^2^ − 28.493X + 13.790	0.755	170.884	<0.001
Sandy loam A	0%	Y= −2.382X^3^ + 16.458X^2^ − 10.868X + 2.146	0.908	479.697	<0.001
	20%	Y = 0.504X^3^ − 3.199X^2^ + 25.842X − 18.401	0.932	662.438	<0.001
	40%	Y = 0.589X^3^ − 3.254X^2^ + 23.838X − 17.694	0.893	408.135	<0.001
	60%	Y= −0.411X^3^ + 3.213X^2^ + 10.530X − 9.326	0.895	385.249	<0.001
Sandy loam B	0%	Y= −3.300X^3^ + 23.576X^2^ − 24.736X + 12.995	0.870	391.462	<0.001
	20%	Y= −2.978X^3^ + 23.037X^2^ − 29.246X + 13.929	0.866	358.989	<0.001
	40%	Y= −1.730X^3^ + 13.852X^2^ − 9.944X + 1.555	0.863	347.975	<0.001
	60%	Y= −3.748X^3^ + 30.246X^2^ − 49.957X + 28.646	0.840	290.376	<0.001

## Data Availability

Data are provided within the article.
